# Supplementary material for: The trouble with triples: Examining the impact of measurement error in mediation analysis
Source: Genetics. 2023 Mar 18;224(1):iyad045. doi: 10.1093/genetics/iyad045 (PMC10158839; doi:10.1093/genetics/iyad045)
Supplement: iyad045_Supplementary_Data [file iyad045_supplementary_data.pdf]

The Trouble with Triples: Examining the Impact of Measurement Error in Mediation Analysis

Supplemental Materials

Figure S1 Classifications of simulated data with increasing sample sizes. Percent of data sets assigbned to each of the three causal structures (colored line) is shown as a function of sample size. Columns correspond to the simulated causal structure. Classification rates are shown for three-choice (top row) and expanded (bottom row) model options. The correct model is denoted with triangles and the incorrect models are denoted with circles.

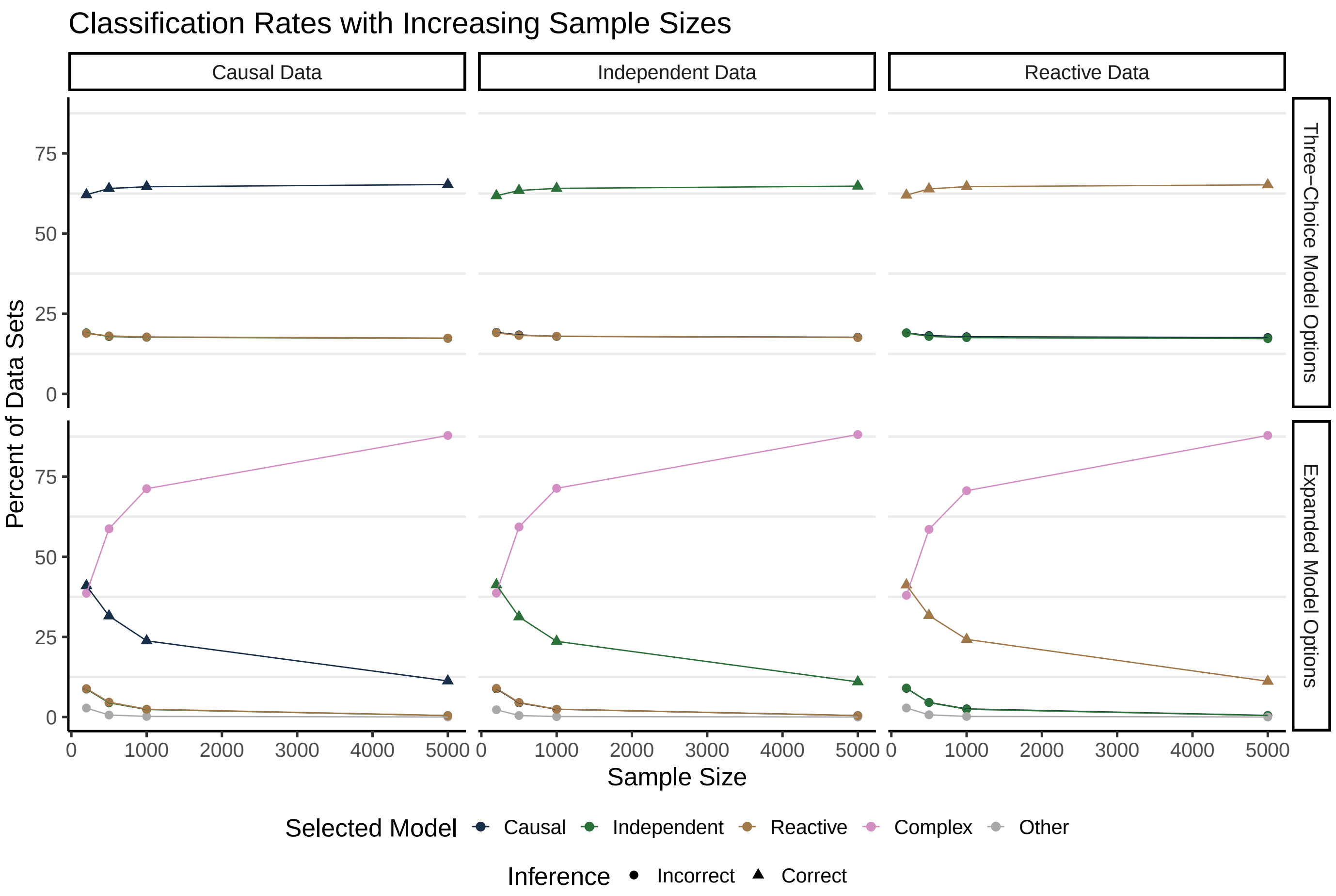

Figure S2 Estimated data correlations from simulations with $N = 5000$ and expanded model options. Each row of panels corresponds to simulations of a causal structure. Columns correspond to binned values of r_{YM}. The x- and y-axes show r_{XM} and r_{XY}, respectively. Points representing the estimated data correlations are colored to indicate the model with the greatest posterior probability. Shaded regions indicate the three-choice model selection inference rule, and the unshaded region delineates where the correlation matrices are not positive semi-definite. See Figure 4 in the main text for results from simulations with $N = 200$.

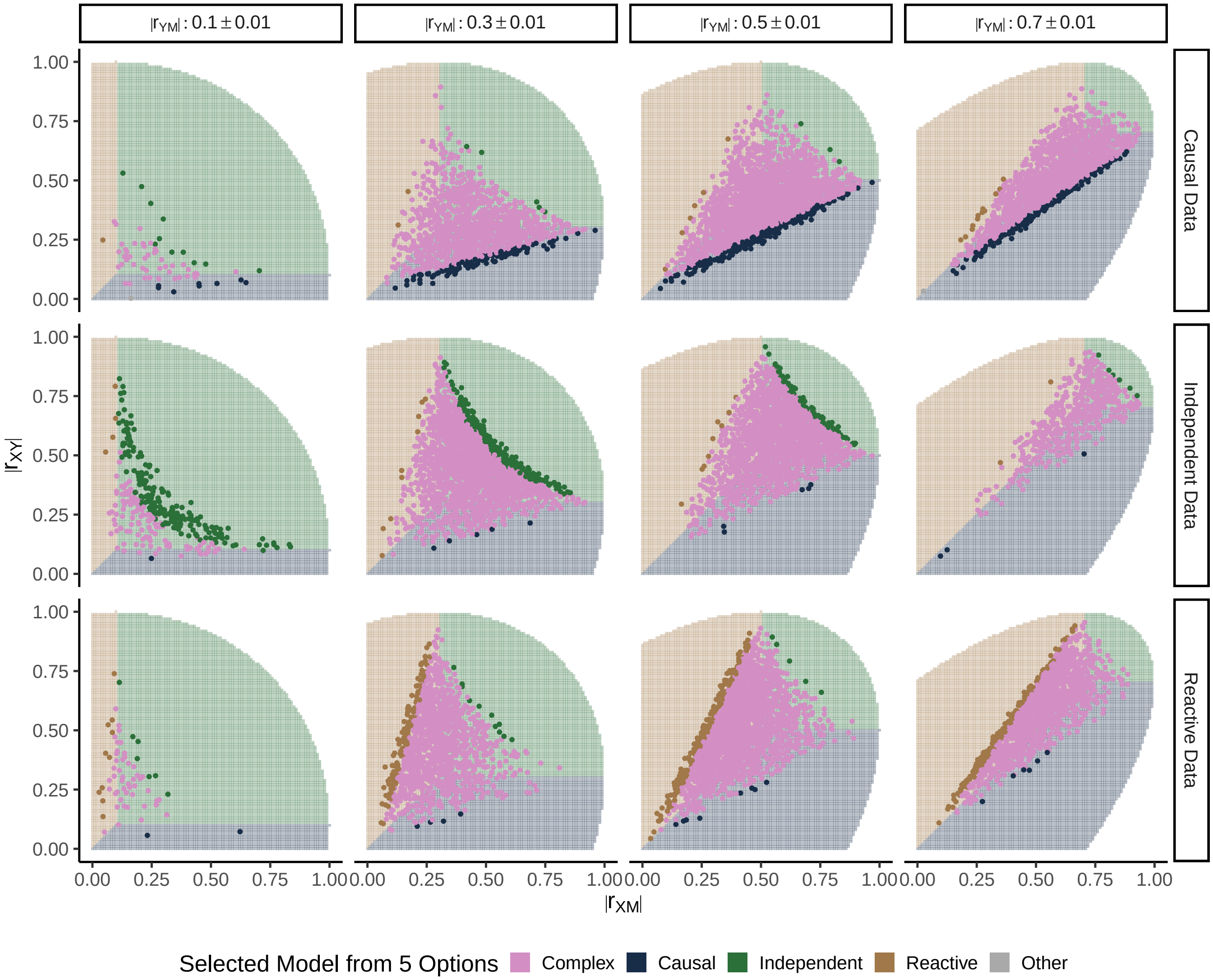

Figure S3 Latent variable representation of the Independent measurement error model. Configurations of the latent variable model representing the Independent model that result in (A) consistent and (B-C) inconsistent inferences. Figure designed the same way as Figure 6 in the main text. (A) The correct model is inferred if the latent correlation ρ_{XU} is the strongest (the latent variable arm for X is the shortest). This can be achieved if there is an equal amount of error in all three variables (top right) or if there is less error in X than M and Y (middle left). If X is noisier than M and Y, the correct model may still be inferred if the causal correlations are weak (middle right). The bottom row shows scenarios where M and Y satisfy different configurations. (B) The Causal model is inferred if ρ_{MU} is the strongest. When the causal structure is the Independent model, this will only occur if ρ_{XU} is weaker than both the error correlation and causal correlation contributing to ρ_{MU}. The relative magnitude of the causal and error components of ρ_{MU} do not matter as long as their product results in the strongest latent correlation. Similarly, the relative magnitude of the causal and error components of ρ_{YU} do not matter as long as ρ_{YU} is not larger than ρ_{MU}. (C) Shows the analogous scenario to (B) for inferring the Reactive model by swapping M and Y.

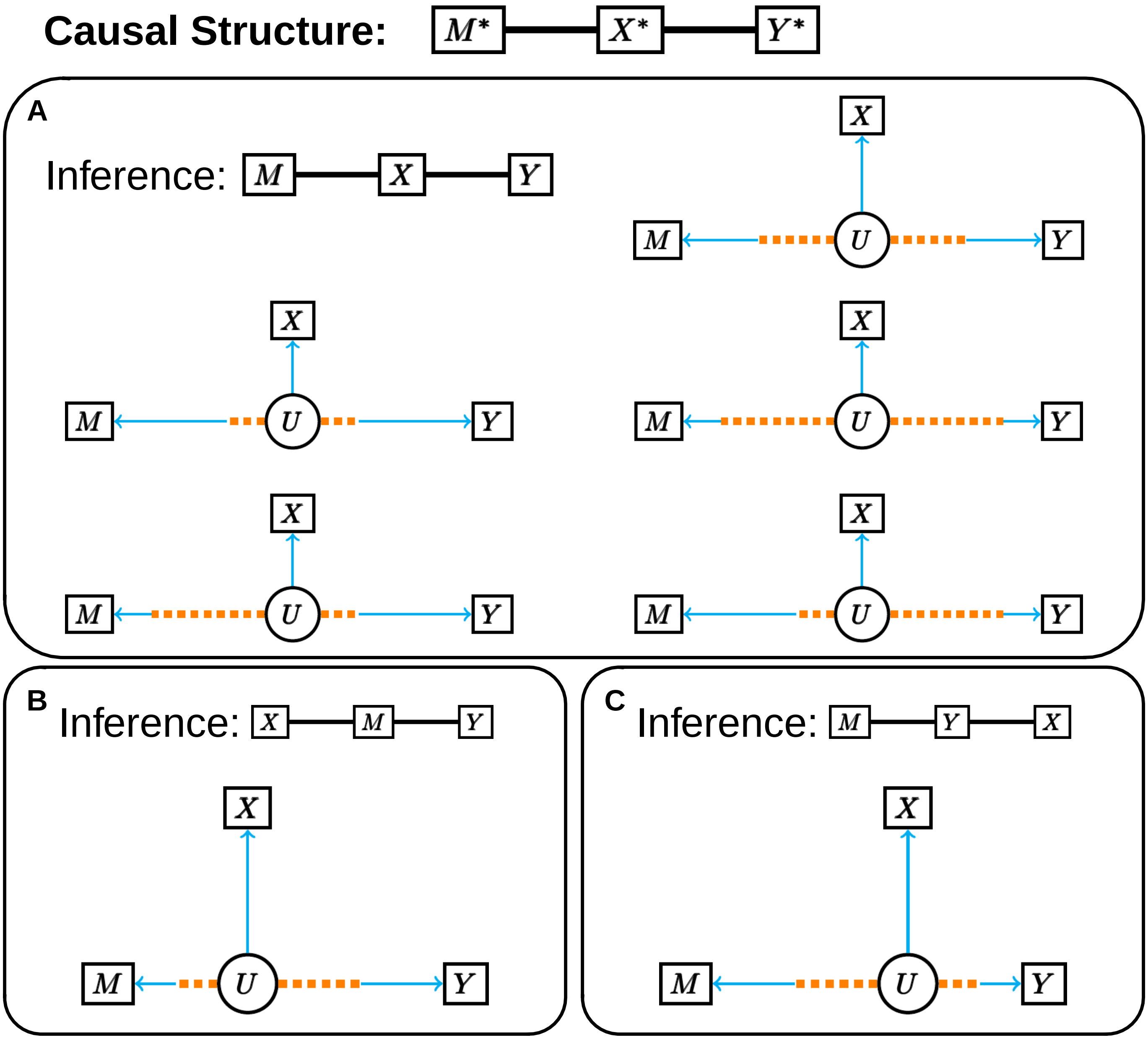

Figure S4 Latent variable representation of the Reactive measurement error model. Configurations of the latent variable model representing the Reactive model that result in (A) consistent and (B-C) inconsistent inferences. Figure designed the same way as Figure 6 in the main text. (A) The correct model is inferred if the latent correlation for Y is the strongest (the latent variable arm for Y is the shortest). This can be achieved if there is an equal amount of error in all three variables (top right) or if there is less error in Y than X and M (middle left). If Y is noisier than X and M, the correct model may still be inferred if the causal correlations are weak (middle right). The bottom row shows scenarios where X and M satisfy different configurations. (B) The Independent model is inferred if ρ_{XU} is strongest. When the causal structure is the Reactive model, this will only occur if ρ_{YU} is weaker than both the error correlation and causal correlation contributing to ρ_{XU}. The relative magnitude of the causal and error components of ρ_X do not matter as long as their product results in the strongest latent correlation. Similarly, the relative magnitude of the causal and error components of ρ_{MU} do not matter as long as ρ_{MU} is not larger than ρ_{XU}. (C) Shows the analogous scenario to (B) for inferring the Causal model by swapping X and M.

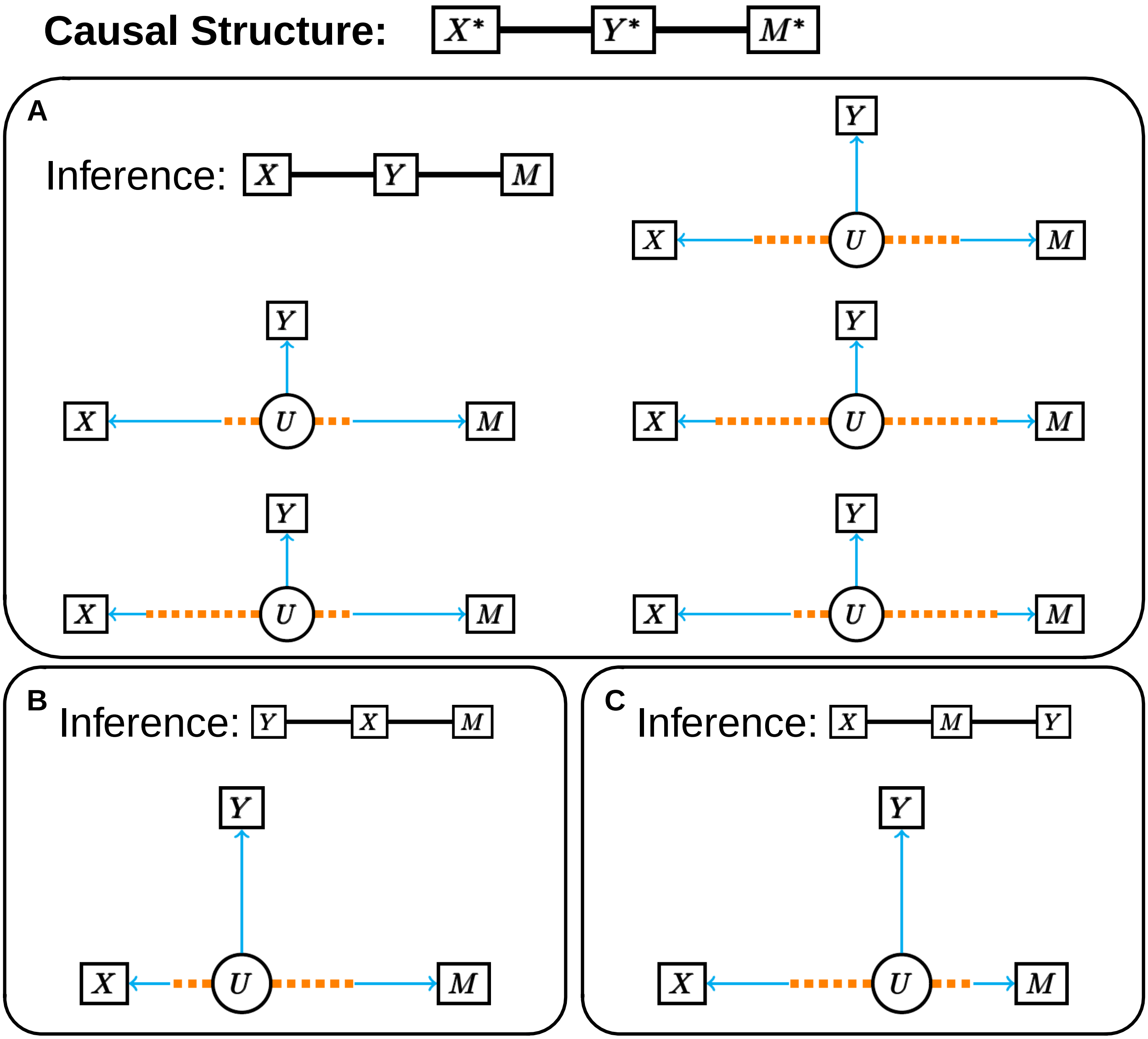

Figure S5 Latent correlations estimated from observed data. (A) Estimated latent correlations for a subset of 1000 simulations of the Causal measurement error model with univariate X. (B) Estimated latent correlations for simulations of the same measurement error models using multi-state X randomly selected from the genotype probabilites from liver tissue of 835 DO mice.

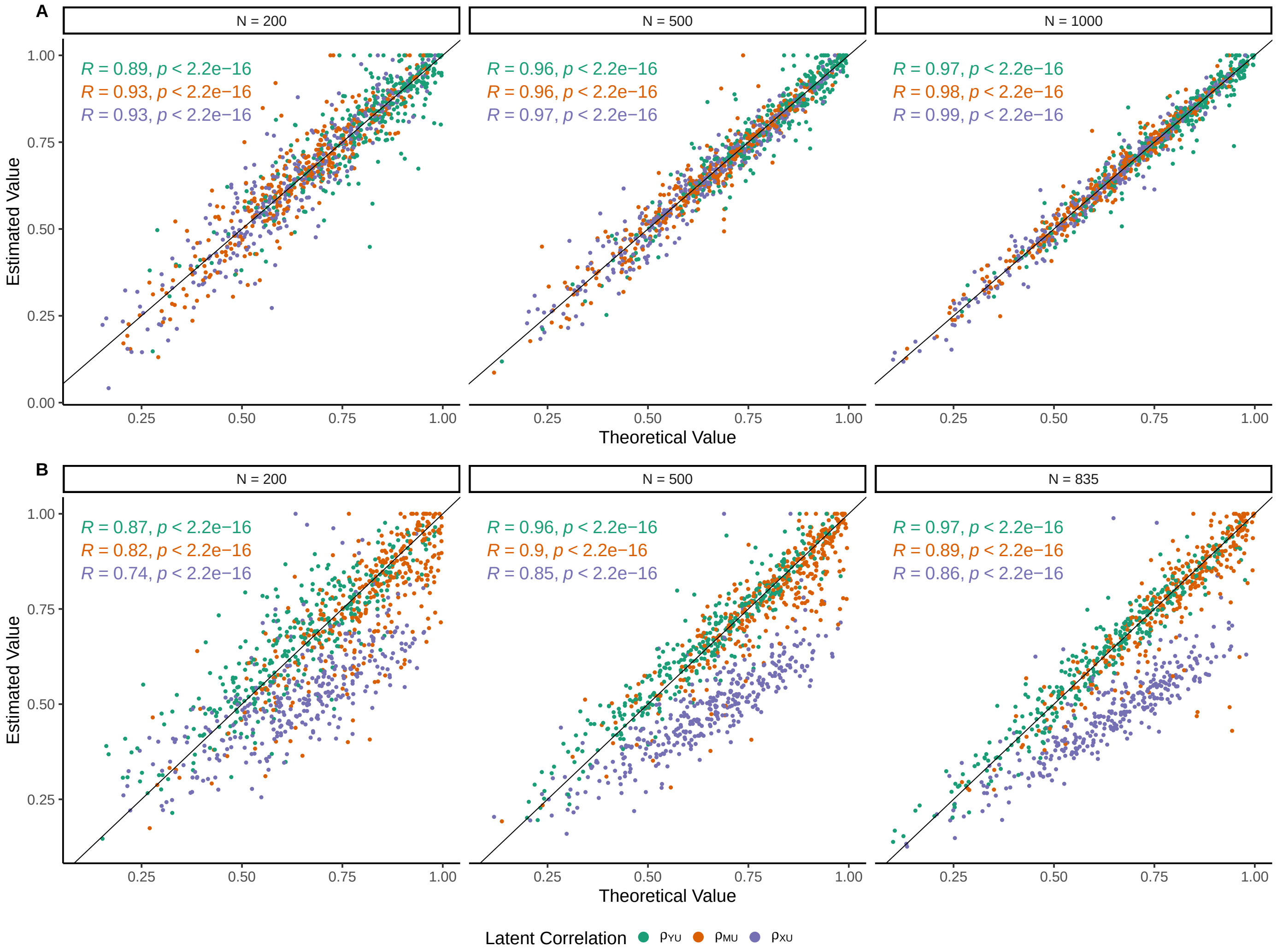

Table S1 Measurement Error Model Configurations. Configurations of the measurement error model that will lead to consistent inferences (diagonal boxes) versus inconsistent inferences (off-diagonal boxes) when classified with three-choice model options.

Causal Structure	Selected Model		
	**Causal**	**Independent**	**Reactive**
**Causal**	Either $\rho_{M^*M} \geq \rho_{X^*X}$ or<br>$\rho_{M^*M} < \rho_{X^*X}$ & $\rho_{X^*M^*} << 1$ st.<br>$\rho_{M^*M} > \rho_{X^*X} \cdot \rho_{X^*M^*}$<br>and<br>Either $\rho_{M^*M} \geq \rho_{Y^*Y}$ or<br>$\rho_{M^*M} < \rho_{Y^*Y}$ & $\rho_{Y^*M^*} << 1$ st.<br>$\rho_{M^*M} > \rho_{Y^*Y} \cdot \rho_{Y^*M^*}$	$\rho_{M^*M} << \rho_{X^*X}$ and<br>$\rho_{M^*M} << \rho_{X^*M^*}$ st.<br>$\rho_{M^*M} < \rho_{X^*X} \cdot \rho_{X^*M^*}$<br>and<br>$\rho_{X^*X} \cdot \rho_{X^*M^*} > \rho_{Y^*Y} \cdot \rho_{Y^*M^*}$	$\rho_{M^*M} << \rho_{Y^*Y}$ and<br>$\rho_{M^*M} << \rho_{Y^*M^*}$ st.<br>$\rho_{M^*M} < \rho_{Y^*Y} \cdot \rho_{Y^*M^*}$<br>and<br>$\rho_{Y^*Y} \cdot \rho_{Y^*M^*} > \rho_{X^*X} \cdot \rho_{X^*M^*}$
**Independent**	$\rho_{X^*X} << \rho_{M^*M}$ and<br>$\rho_{X^*X} << \rho_{X^*M^*}$ st.<br>$\rho_{X^*X} < \rho_{M^*M} \cdot \rho_{X^*M^*}$<br>and<br>$\rho_{M^*M} \cdot \rho_{X^*M^*} > \rho_{Y^*Y} \cdot \rho_{X^*Y^*}$	Either $\rho_{X^*X} \geq \rho_{M^*M}$ or<br>$\rho_{X^*X} < \rho_{M^*M}$ & $\rho_{X^*M^*} << 1$ st.<br>$\rho_{X^*X} > \rho_{M^*M} \cdot \rho_{X^*M^*}$<br>and<br>Either $\rho_{X^*X} \geq \rho_{Y^*Y}$ or<br>$\rho_{X^*X} < \rho_{Y^*Y}$ & $\rho_{X^*Y^*} << 1$ st.<br>$\rho_{X^*X} > \rho_{Y^*Y} \cdot \rho_{X^*Y^*}$	$\rho_{X^*X} << \rho_{Y^*Y}$ and<br>$\rho_{X^*X} << \rho_{X^*Y^*}$ st.<br>$\rho_{X^*X} < \rho_{Y^*Y} \cdot \rho_{X^*Y^*}$<br>and<br>$\rho_{Y^*Y} \cdot \rho_{X^*Y^*} > \rho_{M^*M} \cdot \rho_{X^*M^*}$
**Reactive**	$\rho_{Y^*Y} << \rho_{M^*M}$ and<br>$\rho_{Y^*Y} << \rho_{Y^*M^*}$ st.<br>$\rho_{Y^*Y} < \rho_{M^*M} \cdot \rho_{Y^*M^*}$<br>and<br>$\rho_{M^*M} \cdot \rho_{Y^*M^*} > \rho_{X^*X} \cdot \rho_{X^*Y^*}$	$\rho_{Y^*Y} << \rho_{X^*X}$ and<br>$\rho_{Y^*Y} << \rho_{X^*Y^*}$ st.<br>$\rho_{Y^*Y} < \rho_{X^*X} \cdot \rho_{X^*Y^*}$<br>and<br>$\rho_{X^*X} \cdot \rho_{X^*Y^*} > \rho_{M^*M} \cdot \rho_{Y^*M^*}$	Either $\rho_{Y^*Y} \geq \rho_{M^*M}$ or<br>$\rho_{Y^*Y} < \rho_{M^*M}$ & $\rho_{Y^*M^*} << 1$ st.<br>$\rho_{Y^*Y} > \rho_{M^*M} \cdot \rho_{Y^*M^*}$<br>and<br>Either $\rho_{Y^*Y} \geq \rho_{X^*X}$ or<br>$\rho_{Y^*Y} < \rho_{X^*X}$ & $\rho_{X^*Y^*} << 1$ st.<br>$\rho_{Y^*Y} > \rho_{X^*X} \cdot \rho_{X^*Y^*}$
